# Design of the EXercise Intervention after Stem cell Transplantation (EXIST) study: a randomized controlled trial to evaluate the effectiveness and cost-effectiveness of an individualized high intensity physical exercise program on fitness and fatigue in patients with multiple myeloma or (non-) Hodgkin's lymphoma treated with high dose chemotherapy and autologous stem cell transplantation

**DOI:** 10.1186/1471-2407-10-671

**Published:** 2010-12-06

**Authors:** Saskia Persoon, Marie José Kersten, Mai JM ChinAPaw, Laurien M Buffart, Heleen Burghout, Goof Schep, Johannes Brug, Frans Nollet

**Affiliations:** 1 Department of Rehabilitation, Academic Medical Center, University of Amsterdam, Amsterdam, the Netherlands; 2 Department of Hematology, Academic Medical Center, University of Amsterdam, Amsterdam, the Netherlands; 3 EMGO Institute for Health and Care Research, department of Public and Occupational Health, VU University Medical Center, Amsterdam, the Netherlands; 4 EMGO Institute for Health and Care Research, department of Epidemiology and Biostatistics, VU University Medical Center, Amsterdam, the Netherlands; 5 FysioAlign, Ede, the Netherlands; 6 Department of Sports Medicine, Maxima Medical Center, Veldhoven, the Netherlands

## Abstract

**Background:**

The use of high-dose chemotherapy combined with autologous stem cell transplantation has improved the outcome of hematologic malignancies. Nevertheless, this treatment can cause persistent fatigue and a reduced global quality of life, role and physical function. Physical exercise interventions may be beneficial for physical fitness, fatigue and quality of life. However, the trials conducted so far to test the effects of physical exercise interventions in this group of patients were of poor to moderate methodological quality and economic evaluations are lacking. Hence there is need for a rigorous, appropriately controlled assessment of the effectiveness of exercise programs in these patients. The aims of the present study are (1) to determine the effectiveness of an individualized high intensity strength and interval training program with respect to physiological and psychological health status in patients with multiple myeloma or (non-)Hodgkin's lymphoma who have recently undergone high dose chemotherapy followed by autologous stem cell transplantation; and (2) to evaluate the cost-effectiveness of this program.

**Methods:**

A multicenter, prospective, single blind randomized controlled trial will be performed. We aim to recruit 120 patients within an inclusion period of 2 years at 7 hospitals in the Netherlands. The patients will be randomly assigned to one of two groups: (1) intervention plus usual care; or (2) usual care. The intervention consists of an 18-week individualized supervised high-intensity exercise program and counselling. The primary outcomes (cardiorespiratory fitness, muscle strength and fatigue) and secondary outcomes are assessed at baseline, at completion of the intervention and at 12 months follow-up.

**Discussion:**

The strengths of this study include the solid trial design with clearly defined research groups and standardized outcome measures, the inclusion of an economic evaluation and the inclusion of both resistance and endurance exercise in the intervention program.

**Trial registration:**

This study is registered at the Netherlands Trial Register (NTR2341)

## Background

The use of high-dose chemotherapy (HDC) combined with autologous stem cell transplantation (ASCT) has improved the outcome of haematological malignancies such as multiple myeloma (MM) and (non-)Hodgkin's lymphoma (NHL/HL). It has become standard of care in these diseases in first line and relapse, respectively. However, this treatment has long term negative side effects. Symptoms like fatigue [1-7] and dyspnoea [1,4,5,8] are highly prevalent among ASCT survivors. In addition, survivors have a reduced global quality of life, role and physical function when compared to population norms [9]. For instance, up to 60% of the patients 3 years post-transplant had a compromised ability to engage in activities as carrying a heavy bag and taking a long walk [10]. 23-56% of the patients were not able to return to work during the course of the first year after ASCT [6,11-13].

The persistent fatigue and deficits in health related quality of life (HRQoL) might reflect a self-perpetuating condition [14-18]. Cancer, its treatment and the associated bed rest can lead to poor physical fitness (a.o. impaired cardiorespiratory function and reduced muscle strength). As a result, greater effort is required to fulfil the activities of daily living, and performance of these activities can induce an abnormally high level of fatigue. In order to minimize fatigue, patients will limit physical activities, which will eventually lead to a greater decline in physical fitness. An exercise intervention might break this downward sequence [14-18]. Previous studies have shown that exercise intervention programs can improve physical fitness, fatigue level and quality of life among haematological cancer patients [14,15,17,19-25]. However, based on systematic reviews, Liu et al. (2009) and Wiskemann & Huber (2008) conclude that more high quality research is necessary [26,27]. In the review of Liu et al. (2009) [26] only three of the ten included studies were randomized controlled trials. The overall quality of many studies reviewed was limited, with shortcomings related to trial design, sample size, choice of comparison groups, outcome measures and duration of follow up [26]. Wiskemann & Huber (2008) reached similar conclusions [27]. Both reviews show that there is a need for well designed, randomized controlled trials that verify the findings of the previous trials and can lead to evidence-based interventions.

In addition to the limited methodological quality, the trials performed to date were heterogeneous in terms of the type of exercise interventions studied. Most of the studies focussed on isolated aerobic exercise during or after the stem cell transplantation. Resistance exercise programs and combined training strategies have been evaluated more rarely [27]. This is somewhat surprising since muscle atrophy is a common problem in cancer patients [28-30]. The muscle athrophy is likely to be even more pronounced in patients undergoing HDC and ASCT because of the nature of drugs being used (a.o. high dose glucocorticoids) [16], and because of the morbidity associated with the neutropenic phase after ASCT, which often leads to prolonged bed rest. As considerable evidence suggests that the ability to perform physical tasks in daily life is determined by a certain threshold level of muscular strength [31], it seems important that exercise interventions not only aim to improve aerobic capacity but also aim to minimize muscle atrophy or even stimulate muscle hypertrophy.

To our knowledge, there are currently no data on the cost-effectiveness of exercise intervention programs in cancer patients. The common inability to return to work during the course of the first year after ASCT [6,11-13], the frequent use of health care resources [32,33] and the reported financial problems by patients [8,34] show the importance of determining the cost-effectiveness of exercise intervention programs.

The current study will evaluate an individualized high intensity strength and interval training program developed and pilot-tested by De Backer et al. (2007, 2008) at the Maxima Medical Center (MMC) in Veldhoven, the Netherlands [35,36]. This program has shown promising results with respect to rehabilitation of cancer patients after chemotherapy, but needs to be further explored and tested in ASCT survivors.

The aims of the current study are (1) to determine the effectiveness of a state-of-the-art individualized high intensity strength and interval training program with respect to physiological and psychological status in patients with MM, NHL or HL who have recently undergone HDC followed by ASCT; and (2) to evaluate the cost-effectiveness of this exercise program.

We hypothesize that this exercise program will lead to (1) improved physical fitness; (2) lower levels of fatigue; (3) less mood disturbance; (4) higher levels of daily activities; (5) improved HRQoL; (6) a higher partial and full return to work rate; and that the program (7) will be cost-effective when compared to standard care only.

## Methods

The EXIST (EXercise Intervention after Stem cell Transplantation) study is one of four randomised controlled trials included in the Alpe d'HuZes Cancer Rehabilitation (A-CaRe) program.

This study consists of a pilot study which is followed by a multicenter, prospective, single blind randomized controlled trial (RCT). The protocol of the pilot study will be similar to the RCT protocol described in this manuscript, with the exception of the long term follow up. The aims of this pilot study are to evaluate the feasibility of the intervention and to test the study logistics. For these aims, the patients will also be interviewed during and after completion of the program about: (1) the perceived efficacy of and satisfaction with the intervention program and (2) the need for changes to the program. If necessary fine tuning of the intervention will take place.

**Figure 1:**
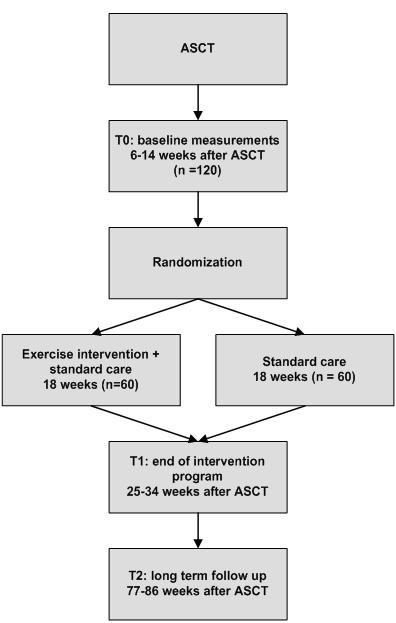
**Scheme of the study**. ASCT: autologous stem cell transplantation.

The aim of the RCT is to compare the exercise intervention and usual care with usual care only (Figure 1). Eligible patients will be randomly assigned to the intervention or control group. In addition to usual care, patients in the intervention group will take part in an 18-weeks individualized supervised high-intensity exercise program. This program will start 7-14 weeks after ASCT. Patients in the control group are treated according to usual care. The study protocol was approved by the Medical Ethics Committee of the Academic Medical Center (METC AMC 10/106).

### Study sample

Eligibility criteria are: (1) diagnosed with MM in first line or with HL/NHL in first relapse and treated with HDC and ASCT 6 to 12 weeks ago; (2) sufficiently recovered from the ASCT: Hb > 6.5 mmol/L, WBC > 3.0 × 10^9^/L, platelets > 100 × 10^9^/L; (3) aged between 18 and 65 years; (4) able to cycle on a bicycle ergometer with a load of at least 25 Watt; (5) able to walk at least 100 meters independently without crutches/cane(s) or walking frame; (6) give written informed consent. Exclusion criteria are (1) treatment with autologous-allogeneic SCT; presence of (2) severe cognitive impairment; (3) severe emotional instability; (4) extensive osteolytic lesions with risk of fracture; (5) serious cardiorespiratory and/or cardiovascular conditions; (6) severe infections; (7) progression/relapse of the disease; (8) other disabling comorbidity interfering with the intervention program or influencing outcome parameters (a.o. having a pacemaker, epileptic seizures and/or poorly regulated diabetes mellitus); (9) insufficient mastery of the Dutch language.

### Recruitment and randomization

Our goal is to recruit 120 patients within an inclusion period of two years. Recruitment takes place at the Academic Medical Center (AMC; Amsterdam), Antoni van Leeuwenhoek Hospital (Amsterdam), Haga Hospital (Den Haag), Meander Medical Center (Amersfoort), Onze Lieve Vrouwe Gasthuis (Amsterdam), St. Antonius Hospital (Nieuwegein) and University Medical Center Utrecht. All potentially eligible patients are asked to complete a short screening questionnaire and are informed about EXIST by their treating haematologist. This screening questionnaire assesses co-morbidity, pre-illness lifestyle, current attitudes toward and beliefs about exercise in general and exercising after ASCT. These questions are adapted from measures developed by Courneya and colleagues [37,38] for use in evaluating exercise in cancer survivors, and are based on established health behaviour theories, in particular the Theory of Planned Behaviour [38]. Patients who are not willing to participate in EXIST are asked for the reason for non-participation. Patients who are willing to participate are asked to provide written informed consent.

After the baseline measurements a randomization will take place conform a stratified block randomization method with a block size of 4 and by using a validated software package [39]. Stratification factors include transplant center and diagnosis. A member of the research team will inform the patient of the randomization outcome. Study outcomes will be assessed by blinded professionals and patients will be instructed not to reveal their group allocation.

### Intervention

Besides the usual care, patients in the intervention group follow an 18-week exercise program similar to the program developed by De Backer et al. (2007, 2008) [35,36]. This program consists of high-intensity resistance and interval training. Before the start of the intervention (T = 0) a sports physician screens the patients and, adapts the program in case of physical limitations. Training takes place in local physiotherapy practices supervised by physiotherapists. Patients will train on specialized resistance training equipment and bicycle ergometers. Furthermore, the physiotherapist will motivate the patient to maintain an active lifestyle. A detailed training manual will be available. Table 1 presents the structure of the intervention program.

**Table 1:** Structure of the exercise program

Week	Type of training	Number of training sessions	Aims of the training
1-12	Resistance training and interval training (2 × 8 minutes).	2× per week, 60 minutes.	1. become familiar with exercise program; 2. overcoming the fear of physical activity; 3. improve coordination and muscle hypertrophy and hereby improving muscle force; 4. increasing aerobic capacity; 5. increasing the pleasure in being physically active.

13-18	Resistance training and interval training (2 × 8 minutes).	1× per week, 60 minutes.	1. maintain muscle force; 2. improve muscle endurance; 3. improve aerobic capacity.

1, 4, 10, 12, 18, 22	Counseling.	6 sessions of 5 to 15 minutes	1. improve compliance to the exercise intervention; 2. encourage patients to pursue an active lifestyle.

Training: 30 sessions a 60 minutes = 30 hours

Counseling: 6 sessions a 15 minutes = 1.5 hours

#### Resistance exercises

The six resistance exercises target the large muscle groups: (1) vertical row (longissimus, biceps brachii, rhomboideus); (2) leg press (quadriceps, glutei, gastrocnemius); (3) bench press (pectoralis major, triceps); (4) pull over (pectoralis, triceps brachii, deltoideus, trapezius); (5) abdominal crunch (rectus abdominis); (6) lunge (quadriceps, glutei, hamstrings). Indirect one repetition maximum (1-RM) measurements will be performed every four weeks for all six exercises. In the first 12 weeks, resistance exercise consists of two sets of 10 repetitions at 65 to 80% of the 1-RM. From week 12 onwards it comprises more repetitions (20 repetitions per set) at a lower resistance (35-40%).

#### Interval training

Before and after the resistance exercises patients cycle two times eight minutes. To determine the right resistance a steep ramp test [40-42] is performed every four weeks. With this test the subject is instructed to cycle at a speed between 70 and 80 revolutions per minute (RPM), starting at a work load of 25W for 30 seconds. Hereafter the load is increased by 25W every 10 seconds until exhaustion. Maximal short exercise capacity (the maximal workload; MSEC) is recorded. In the first eight weeks, blocks of 30 seconds at 65% of the MSEC will be alternated with blocks of 60 seconds at 30%. From week nine onwards, the duration of the latter block is reduced to 30 seconds.

#### Counselling

A behavioural motivation component is included to improve compliance and to stimulate physical activity outside the exercise program in addition to and after completion of the exercise program. From week 12 onwards patients are encouraged to meet the recommendations made by the American College of Sports Medicine and the American Heart Association [43]. Specific program elements include the provision of general and motivational information, both verbally and via folders, about physical activity and the desired intensity of activity based on the Borg Scale rating perceived exertion [44]. The physiotherapist uses basic counselling techniques and instruction sheets.

### Standard care

Both the patients in the intervention group and the patients in the control group receive 'usual care'. In the Netherlands usual care varies according to doctors' and patients' preferences. Patients in the control group are allowed to participate in sports or existing rehabilitation programs. Information on physical activity and exercise of all patients will be obtained from the physical activity questionnaire, recordings of the accelerometer and cost-diaries (see outcome measures).

### Study outcomes

**Figure 2:**
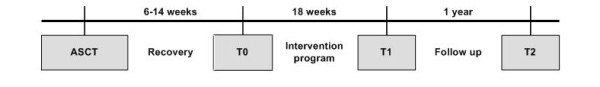
**Timeline of the study**. ASCT: autologous stem cell transplantation; T0: baseline; T1: post treatment; T2: long term follow-up.

All studies within A-CaRe Clinical Research use similar methodologies and a comparable set of outcome measures. Figure 2 shows the timing of the assessments. The physical tests are performed centrally at the AMC according to a detailed and standardized protocol. The questionnaires will be filled out at home.

#### Primary outcomes measures

##### Cardiorespiratory fitness

At rest respiratory function will be assessed by measuring the forced air expiratory volume in 1 second (FEV-1), the forced vital capacity (FVC), inspiratory capacity (IC) and by estimating the maximal voluntary ventilation (MVV) [45,46].

A maximal exercise test will be performed to assess cardiorespiratory fitness and potential cardiovascular and cardiorespiratory limitations. This technique has been shown to be feasible in cancer survivors [18].

We will follow international guidelines concerning standardization and interpretation strategies [47]. The maximal exercise test will be performed on a cycle ergometer (Lode Corival, Groningen, the Netherlands) under supervision of a sports physician. A ramp test design will be applied: after four minutes of unloaded cycling, the load will be gradually increased until exhaustion or inability to maintain pedal frequency above 60 RPM. Throughout the test the ECG, saturation and blood pressure will be monitored and heart rate (HR) and gas exchange variables (MasterScreen CPX, CareFusion, Hoechberg, Germany) will be recorded continuously and averaged at 30 seconds intervals. We will register peak oxygen uptake (peakVO_2_), maximal heart rate (peak HR) and mean power output (peak W). Ventilatory threshold (VT) will be determined using the ventilatory equivalents method [47]. The respiratory exchange ratio (RER) and HR will be used to review peak exercise. After the start of recovery, the patient will be asked about his/her exercise-related perceptions using the Borg Scale of perceived exertion [44].

##### Muscle strength

Upper extremity muscle strength is assessed using a grip strength dynamometer (Hydraulic Hand Dynamometer, North Coast Medical Inc., Morgan Hill, USA), and lower extremity muscle strength with the 30 seconds chair stand test. Maximal handgrip strength is measured following the standard procedures recommended by the American Society of Hand Therapists (ASHT) [48]. The mean score attained for each side will be recorded. The 30 seconds chair stand test has been demonstrated to be a valid and reliable measure of proximal lower limb strength in older adults [49]. The subject is asked to stand upright from a chair with arms crossed at the wrist and held against the chest, then return to a fully seated position and repeat the action at his/her fastest pace over a 30 seconds period. The number of full stands within this time period is recorded.

##### Fatigue

Two self-report questionnaires will be used to assess fatigue: the Multidimensional Fatigue Inventory (MFI) and the Fatigue Quality List (FQL). The MFI [50] is a questionnaire consisting of 20 statements for which the person has to indicate on a 0-5 scale to what extent the particular statement applies to him or her. This self-report instrument consists of five subscales based on different dimensions: general fatigue, physical fatigue, reduced activity, reduced motivation and mental fatigue.

The patients' perception and appraisal of experienced fatigue will be assessed with the FQL [51]. The FQL consists of 25 adjectives describing the fatigue experience, organized into four subscales: frustrating, exhausting, pleasant, and frightening.

#### Secondary outcomes measures and moderating variables

Secondary outcomes are body composition, bone mineral density, HRQoL, neuropathy, objective and self reported physical activity level, mood disturbance, functioning in daily life, return to work and cost from a social perspective. In addition, clinical data, disease status and treatment, sociodemographic characteristics, adverse events and potential predictors of compliance and satisfaction with the exercise program will be recorded. A complete overview of assessments and instruments are presented in Table 2. A small selection of these measures is described in detail here.

**Table 2:** Assessments and instruments

Outcome measures	Instrument
**Primary**	

Cardiorespiratory fitness	Maximal exercise test.

Muscle strength	Maximum handgrip strength and 30 seconds chair stands test.

Fatigue	Multidimensional Fatigue Inventory (MFI) [50] and the Fatigue Quality List (FQL) [51] questionnaires.

**Secondary**	

Body composition and bone mineral density	DXA-scans^3 ^[55,56] and physical examination; height, weight, waist and hip circumferences, four skinfolds (biceps, triceps, suprailiacal and subscapular).

Objective level of physical activity	Recordings of the Actitrainer accelerometer (Actigraph, Fort Walton Beach, USA).

Health-related quality of life	EORTC Quality of Life Questionnaire C30 (EORTC QLQ-C30) [57], the EORTC Myeloma Module (QLQ-MY20) [58].

Neuropathy^3^	EORTC Chemotherapy-induced peripheral neuropathy module (QLQ-CIPN20) [59].

Self reported physical activity	PASE questionnaire [60].

Mood disturbance	Hospital Anxiety and Depression Scale (HADS) questionnaire [61,62].

Functioning in daily life	Impact on Participation and Autonomy (IPA) questionnaire [63].

Return to work	Return to work questionnaire.

Cost questionnaires^4^	EuroQol (EQ5D) [54] and cost questionnaires.

**Other**	

Sociodemographic data^1^	Age, education, marital status, living situation, co-morbidities and life style variables (e.g. smoking).

Medical history^1^	Date of diagnosis, subtype of disease, stage of disease, history of therapy will be recorded from medical records.

Disease status and treatment	Blood levels (incl. platelet and erythrocyte count), response to treatment, progression or relapse of disease and data on any additional treatment will be recorded from medical records.

Comorbidity	A questionnaire and at T = 0 a sports physician will examine comorbidities that might interfere with the intervention program or influence study outcome.

Adverse events	Medical records, reports of the sports physician and physiotherapist.

Potential predictors of compliance	Questionnaire about pre-illness lifestyle, current attitudes toward and beliefs about exercise in general.

Satisfaction with the intervention^2^	Satisfaction questionnaire; intervention arm only.

Compliance with the exercise program^2^	Self-report and objective measures (e.g. attendance, exercise logs, target intensity); intervention arm only.

^1 ^assessment at T = 0 only

^2 ^assessment at T = 1 only

^3 ^assessment at T = 0, T = 2

^4 ^monthly assessments between T = 0 and T = 1 and once every 3 months between T = 1 and T = 2

##### Objectively assessed level of physical activity

The Actitrainer accelerometer (Actigraph, Fort Walton Beach, USA) will be used to measure physical activity. The Actitrainer is able to measure accelerations from 0.05 to 2.00 G. These accelerations are scored in "counts" per minute. The Actitrainer will be set at 60 seconds and the measurement period will include five days including at least one weekend day.

##### Costs from a societal perspective

Costs will be measured from a societal perspective. The following are being considered in this study: (1) Health care costs: the costs of oncological care, general practice care and physiotherapy; additional visits to other health care providers, prescriptions of medication, professional home care and hospitalization. (2) Patient and family costs: out-of-pocket expenses (e.g. travel expenses), costs for sports and sports equipment, and costs of paid and unpaid help. (3) Costs due to loss of production (absenteeism for patients with paid jobs and hours of inactivity for patients without a paid job). These data will be collected though retrospective cost questionnaires administered on a monthly basis during the period between T = 0 and T = 1. After T = 1 the cost questionnaires will be administered once every three months till T = 2. Health care utilization will be valued using Dutch cost prices [52].

##### Return to work

The following indicators of return to work will be measured: (1) Time to partial and to full return to work (meaning number of calendar days between end of treatment and first day at work), (2) time to full return to work corrected for partial return to work, (3) partial and full return of work rate at T = 1 and T = 2. (4) Details on hours worked per week, nature of work, and return to a different job will also be recorded.

##### Adverse Events

All adverse events noticed by treating physicians and/or physiotherapists or mentioned by the participant will be recorded and monitored. The grading of adverse events will be done using the most recent version of the NCI Common Terminology Criteria for Adverse Events, CTCAE version 4. A complete document may be downloaded from [53].

### Power calculations

The sample size calculations were estimated for our primary study outcomes using a two-sided α = 0.05 and a power of 80%. Based on the results of De Backer et al. (2007, 2008) [35,36] and Hayes et al. (2004) [17], we expect a between group difference of 7.5 ± 7 ml/kg/min in the peak oxygen uptake (peakVO_2_); 0.2 ± 0.1 kg in handgrip strength and 3.5 ± 4 points in fatigue (MFI). We need between 25 and 42 subjects per group to detect these differences between the intervention and control group. We expect a drop-out rate of 30%; 15% of the patients who have undergone autologous SCT for MM, HL or NHL have an early relapse within six months and may not be able to complete the study assessments, and 15% may drop out because of other reasons. Consequently, we need to enrol 60 subjects per group.

### Statistical analyses

Differences in baseline characteristics between intervention and control groups will be tested using independent t-tests, Mann Whitney U tests and chi-square tests.

Data will be analyzed on an intention-to-treat basis. Additionally, a per-protocol analysis will be performed.

Scores on the self-report measures of fatigue, mood state and health-related quality of life will be calculated according to published scoring algorithms. Longitudinal regression analysis will be used to assess differences in each outcome measure between the intervention and control group. The two follow-up measurements will be defined as dependent variable and multi-level analysis with three levels will be used, (1) transplant center, (2) time of follow-up measurement (values corresponding with performance at T = 1 and T = 2), (3) individual.

Regression coefficients indicate differences between intervention and control group. Regression models will be adjusted for baseline values, age and gender. Test results are considered significant for *p*-values < 0.05. All analyses will be performed using the statistics program SPSS.

### Cost-effectiveness analysis

Both cost-effectiveness and cost-utility analyses will be performed. The cost-effectiveness ratios will be calculated by dividing the difference between the mean costs of the two treatment groups by the difference in the mean effects of the two treatment groups. This ratio will include the primary outcome measures of the trial. The cost-utility ratio will express the additional costs of the intervention per quality adjusted life year (QALY). Utilities will be measured using the EuroQol ([54] EQ5D) at baseline, at the end of the treatment and at twelve months.

## Discussion

The EXIST study will assess the effectiveness of a high intensity strength and interval training program on physiological and psychological variables in patients with MM, NHL or HL who have recently undergone HDC followed by ASCT. In addition the cost-effectiveness of the program will be determined.

This study has several strengths. Firstly, the trial design is solid; we will conduct a randomised controlled trial among well defined patient groups, using standardized outcome measures, and including a long term follow up to assess the therapeutic sustainability of the program. Secondly, the exercise program is developed based on existing evidence and consists of both strength and endurance exercise. Therefore, the exercise program is considered to have a higher potential to restore muscle mass compared to an endurance exercise program alone, and consequently, we expect an improvement in the physical as well as the psychological health status of ASCT patients. Furthermore, counselling sessions are included to increase motivation and compliance to physical exercise both during and after completion of the intervention. Thirdly, a cost-effectiveness evaluation will give insight in the costs of the program with respect tot the outcomes.

## Competing interests

The authors declare that they have no competing interests.

## Authors' contributions

SP contributed to the study design, is responsible for data collection, analysis and interpretation and wrote the manuscript. MJK, MC, FN and LB contributed to the conception and the design of the study and revised the manuscript. HB has made contributions to the design of the intervention and contacted the participating physiotherapists. GS developed the intervention program at the MMC. JB revised this manuscript critically. All authors have read and approved the final manuscript.

## Pre-publication history

The pre-publication history for this paper can be accessed here:

http://www.biomedcentral.com/1471-2407/10/671/prepub

## References

[B1] Andrykowski MA Greiner CB Altmaier EM Burish TG Antin JH Gingrich R Quality of life following bone marrow transplantation: findings from a multicentre study Br J Cancer 1995 71 1322 1329 7779732 10.1038/bjc.1995.257 PMC2033838

[B2] Gielissen MF Schattenberg AV Verhagen CA Rinkes MJ Bremmers ME Bleijenberg G Experience of severe fatigue in long-term survivors of stem cell transplantation Bone Marrow Transplant 2007 39 595 603 10.1038/sj.bmt.1705624 17369868

[B3] Gulbrandsen N Hjermstad MJ Wisloff F Interpretation of quality of life scores in multiple myeloma by comparison with a reference population and assessment of the clinical importance of score differences Eur J Haematol 2004 72 172 180 10.1046/j.0902-4441.2003.00195.x 14962235

[B4] Hjermstad MJ Knobel H Brinch L Fayers PM Loge JH Holte H A prospective study of health-related quality of life, fatigue, anxiety and depression 3-5 years after stem cell transplantation Bone Marrow Transplant 2004 34 257 266 10.1038/sj.bmt.1704561 15170167

[B5] Knobel H Loge JH Nordoy T Kolstad AL Espevik T Kvaloy S High level of fatigue in lymphoma patients treated with high dose therapy J Pain Symptom Manage 2000 19 446 456 10.1016/S0885-3924(00)00144-5 10908825

[B6] Lee SJ Fairclough D Parsons SK Soiffer RJ Fisher DC Schlossman RL Recovery after stem-cell transplantation for hematologic diseases J Clin Oncol 2001 19 242 252 11134219 10.1200/JCO.2001.19.1.242

[B7] Andrykowski MA Carpenter JS Greiner CB Altmaier EM Burish TG Antin JH Energy level and sleep quality following bone marrow transplantation Bone Marrow Transplant 1997 20 669 679 10.1038/sj.bmt.1700949 9383231

[B8] Hjermstad M Holte H Evensen S Fayers P Kaasa S Do patients who are treated with stem cell transplantation have a health-related quality of life comparable to the general population after 1 year? Bone Marrow Transplant 1999 24 911 918 10.1038/sj.bmt.1701998 10516705

[B9] Pidala J Anasetti C Jim H Health-related quality of life following haematopoietic cell transplantation: patient education, evaluation and intervention Br J Haematol 2010 148 373 385 10.1111/j.1365-2141.2009.07992.x 19919651 PMC2810350

[B10] Gulbrandsen N Hjermstad MJ Wisloff F Interpretation of quality of life scores in multiple myeloma by comparison with a reference population and assessment of the clinical importance of score differences Eur J Haematol 2004 72 172 180 10.1046/j.0902-4441.2003.00195.x 14962235

[B11] Chao NJ Tierney DK Bloom JR Long GD Barr TA Stallbaum BA Dynamic assessment of quality of life after autologous bone marrow transplantation Blood 1992 80 825 830 1638031

[B12] Hensel M Egerer G Schneeweiss A Goldschmidt H Ho AD Quality of life and rehabilitation in social and professional life after autologous stem cell transplantation Ann Oncol 2002 13 209 217 10.1093/annonc/mdf031 11885996

[B13] Wong FL Francisco L Togawa K Bosworth A Gonzales M Hanby C Long-term recovery after hematopoietic cell transplantation: predictors of quality-of-life concerns Blood 2010 115 2508 2519 10.1182/blood-2009-06-225631 20089962 PMC2845903

[B14] Dimeo F Schwartz S Fietz T Wanjura T Boning D Thiel E Effects of endurance training on the physical performance of patients with hematological malignancies during chemotherapy Support Care Cancer 2003 11 623 628 10.1007/s00520-003-0512-2 12942360

[B15] Dimeo FC Tilmann MH Bertz H Kanz L Mertelsmann R Keul J Aerobic exercise in the rehabilitation of cancer patients after high dose chemotherapy and autologous peripheral stem cell transplantation Cancer 1997 79 1717 1722 10.1002/(SICI)1097-0142(19970501)79:9<1717::AID-CNCR12>3.0.CO;2-0 9128987

[B16] Dimeo FC Effects of exercise on cancer-related fatigue Cancer 2001 92 1689 1693 10.1002/1097-0142(20010915)92:6+<1689::AID-CNCR1498>3.0.CO;2-H 11598888

[B17] Hayes SC Davies PS Parker TW Bashford J Green A Role of a mixed type, moderate intensity exercise programme after peripheral blood stem cell transplantation Br J Sports Med 2004 38 304 309 10.1136/bjsm.2002.003632 15155433 PMC1724811

[B18] Lucia A Earnest C Perez M Cancer-related fatigue: can exercise physiology assist oncologists? Lancet Oncol 2003 4 616 625 10.1016/S1470-2045(03)01221-X 14554239

[B19] Coleman EA Coon S Hall-Barrow J Richards K Gaylor D Stewart B Feasibility of exercise during treatment for multiple myeloma Cancer Nurs 2003 26 410 419 10.1097/00002820-200310000-00012 14710804

[B20] Dimeo F Bertz H Finke J Fetscher S Mertelsmann R Keul J An aerobic exercise program for patients with haematological malignancies after bone marrow transplantation Bone Marrow Transplant 1996 18 1157 1160 8971388

[B21] Dimeo F Rumberger BG Keul J Aerobic exercise as therapy for cancer fatigue Med Sci Sports Exerc 1998 30 475 478 9565925 10.1097/00005768-199804000-00001

[B22] Dimeo FC Stieglitz RD Novelli-Fischer U Fetscher S Keul J Effects of physical activity on the fatigue and psychologic status of cancer patients during chemotherapy Cancer 1999 85 2273 2277 10.1002/(SICI)1097-0142(19990515)85:10<2273::AID-CNCR24>3.0.CO;2-B 10326708

[B23] Hayes S Davies PS Parker T Bashford J Newman B Quality of life changes following peripheral blood stem cell transplantation and participation in a mixed-type, moderate-intensity, exercise program Bone Marrow Transplant 2004 33 553 558 10.1038/sj.bmt.1704378 14716346

[B24] Mello M Tanaka C Dulley FL Effects of an exercise program on muscle performance in patients undergoing allogeneic bone marrow transplantation Bone Marrow Transplant 2003 32 723 728 10.1038/sj.bmt.1704227 13130321

[B25] Wilson RW Jacobsen PB Fields KK Pilot study of a home-based aerobic exercise program for sedentary cancer survivors treated with hematopoietic stem cell transplantation Bone Marrow Transplant 2005 35 721 727 10.1038/sj.bmt.1704815 15696182

[B26] Liu RD Chinapaw MJ Huijgens PC van Mechelen W Physical exercise interventions in haematological cancer patients, feasible to conduct but effectiveness to be established: a systematic literature review Cancer Treat Rev 2009 35 185 192 10.1016/j.ctrv.2008.09.008 19004560

[B27] Wiskemann J Huber G Physical exercise as adjuvant therapy for patients undergoing hematopoietic stem cell transplantation Bone Marrow Transplant 2008 41 321 329 10.1038/sj.bmt.1705917 18026154

[B28] Argiles JM Busquets S Felipe A Lopez-Soriano FJ Molecular mechanisms involved in muscle wasting in cancer and ageing: cachexia versus sarcopenia Int J Biochem Cell Biol 2005 37 1084 1104 10.1016/j.biocel.2004.10.003 15743680

[B29] Tisdale MJ Cachexia in cancer patients Nat Rev Cancer 2002 2 862 871 10.1038/nrc927 12415256

[B30] Tisdale MJ Mechanisms of cancer cachexia Physiol Rev 2009 89 381 410 10.1152/physrev.00016.2008 19342610

[B31] Brill PA Macera CA Davis DR Blair SN Gordon N Muscular strength and physical function Med Sci Sports Exerc 2000 32 412 416 10.1097/00005768-200002000-00023 10694125

[B32] Freeman M Vose J Bennett C Anderson J Kessinger A Turner K Costs of care associated with high-dose therapy and autologous transplantation for non-Hodgkin's lymphoma: results from the University of Nebraska Medical Center 1989 to 1995 Bone Marrow Transplant 1999 24 679 684 10.1038/sj.bmt.1701949 10490736

[B33] Ghatnekar O Alvegard T Conradi N Lenhoff S Mellqvist UH Persson U Direct hospital resource utilization and costs of treating patients with multiple myeloma in Southwest Sweden: a 5-year retrospective analysis Clin Ther 2008 30 1704 1713 10.1016/j.clinthera.2008.09.003 18840377

[B34] Knobel H Loge JH Nordoy T Kolstad AL Espevik T Kvaloy S High level of fatigue in lymphoma patients treated with high dose therapy J Pain Symptom Manage 2000 19 446 456 10.1016/S0885-3924(00)00144-5 10908825

[B35] De Backer I Van Breda E Vreugdenhil A Nijziel MR Kester AD Schep G High-intensity strength training improves quality of life in cancer survivors Acta Oncol 2007 46 1143 1151 10.1080/02841860701418838 17851864

[B36] De Backer I Vreugdenhil G Nijziel MR Kester AD Van Breda E Schep G Long-term follow-up after cancer rehabilitation using high-intensity resistance training: persistent improvement of physical performance and quality of life Br J Cancer 2008 99 30 36 10.1038/sj.bjc.6604433 18577993 PMC2453017

[B37] Courneya KS Understanding readiness for regular physical activity in older individuals: an application of the theory of planned behavior Health Psychol 1995 14 80 87 10.1037/0278-6133.14.1.80 7737078

[B38] Courneya KS Friedenreich CM Utility of the theory of planned behavior for understanding exercise during breast cancer treatment Psychooncology 1999 8 112 122 10.1002/(SICI)1099-1611(199903/04)8:2<112::AID-PON341>3.0.CO;2-L 10335555

[B39] TENALEA Clinical Trial Data Management System. An online, central randomisation service, currently in deployment phase with a grant from the e-TEN programme of the European Union (LSHC-CT-510736) 2010 Ref Type: Computer Program

[B40] De Backer IC Schep G Hoogeveen A Vreugdenhil G Kester AD Van Breda E Exercise testing and training in a cancer rehabilitation program: the advantage of the steep ramp test Arch Phys Med Rehabil 2007 88 610 616 10.1016/j.apmr.2007.02.013 17466730

[B41] Meyer K Samek L Schwaibold M Westbrook S Hajric R Beneke R Interval training in patients with severe chronic heart failure: analysis and recommendations for exercise procedures Med Sci Sports Exerc 1997 29 306 312 9139168 10.1097/00005768-199703000-00004

[B42] Meyer K Exercise training in heart failure: recommendations based on current research Med Sci Sports Exerc 2001 33 525 531 10.1097/00005768-200105001-00874 11283426

[B43] Haskell WL Lee IM Pate RR Powell KE Blair SN Franklin BA Physical activity and public health: updated recommendation for adults from the American College of Sports Medicine and the American Heart Association Circulation 2007 116 1081 1093 10.1161/CIRCULATIONAHA.107.185649 17671237

[B44] Borg GA Psychophysical bases of perceived exertion Med Sci Sports Exerc 1982 14 377 381 7154893

[B45] Miller MR Hankinson J Brusasco V Burgos F Casaburi R Coates A Standardisation of spirometry Eur Respir J 2005 26 319 338 10.1183/09031936.05.00034805 16055882

[B46] Wasserman K Hansen JE Sue DY Stringer WW Whipp BJ Principles of Exercise Testing and Interpretation. Including Pathophysiology and Clinical Applications 2005 fourth Philadelphia: Lippincott Williams & Wilkins

[B47] ERS Task Force on Standardization of Clinical Exercise Testing Clinical exercise testing with reference to lung diseases: indications, standardization and interpretation strategies. European Respiratory Society Eur Respir J 1997 10 2662 2689 10.1183/09031936.97.10112662 9426113

[B48] Fess EE Casanova JS Grip strength Clinical Assessment Recommendations 1992 2 Chicago: American Society of Hand Therapists 41 45

[B49] Rikli RE Jones CE Development and validation of a functional fitness test for community-residing older adults Journal of aging and physical activity 1999 7 129 161

[B50] Smets EM Garssen B Bonke B De Haes JC The Multidimensional Fatigue Inventory (MFI) psychometric qualities of an instrument to assess fatigue J Psychosom Res 1995 39 315 325 10.1016/0022-3999(94)00125-O 7636775

[B51] Gielissen MF Knoop H Servaes P Kalkman JS Huibers MJ Verhagen S Differences in the experience of fatigue in patients and healthy controls: patients' descriptions Health Qual Life Outcomes 2007 5 36 10.1186/1477-7525-5-36 17605783 PMC1934901

[B52] Oostenbrink JB Bouwmans CAM Koopmanschap MA Rutten FFH Handleiding voor kostenonderzoek, methoden en standaard kostprijzen voor economische evaluaties in de gezondheidzorg 2004

[B53] Cancer Therapy Evaluation Program http://ctep.cancer.gov/reporting/ctc.html

[B54] Kopec JA Willison KD A comparative review of four preference-weighted measures of health-related quality of life J Clin Epidemiol 2003 56 317 325 10.1016/S0895-4356(02)00609-1 12767408

[B55] Andreoli A Scalzo G Masala S Tarantino U Guglielmi G Body composition assessment by dual-energy X-ray absorptiometry (DXA) Radiol Med 2009 114 286 300 10.1007/s11547-009-0369-7 19266259

[B56] Blake GM Fogelman I An update on dual-energy x-ray absorptiometry Semin Nucl Med 2010 40 62 73 10.1053/j.semnuclmed.2009.08.001 19958851

[B57] Aaronson NK Ahmedzai S Bergman B Bullinger M Cull A Duez NJ The European Organization for Research and Treatment of Cancer QLQ-C30: a quality-of-life instrument for use in international clinical trials in oncology J Natl Cancer Inst 1993 85 365 376 10.1093/jnci/85.5.365 8433390

[B58] Cocks K Cohen D Wisloff F Sezer O Lee S Hippe E An international field study of the reliability and validity of a disease-specific questionnaire module (the QLQ-MY20) in assessing the quality of life of patients with multiple myeloma Eur J Cancer 2007 43 1670 1678 10.1016/j.ejca.2007.04.022 17574838

[B59] Postma TJ Aaronson NK Heimans JJ Muller MJ Hildebrand JG Delattre JY The development of an EORTC quality of life questionnaire to assess chemotherapy-induced peripheral neuropathy: the QLQ-CIPN20 Eur J Cancer 2005 41 1135 1139 10.1016/j.ejca.2005.02.012 15911236

[B60] Washburn RA Smith KW Jette AM Janney CA The Physical Activity Scale for the Elderly (PASE): development and evaluation J Clin Epidemiol 1993 46 153 162 10.1016/0895-4356(93)90053-4 8437031

[B61] Spinhoven P Ormel J Sloekers PP Kempen GI Speckens AE van Hemert AM A validation study of the Hospital Anxiety and Depression Scale (HADS) in different groups of Dutch subjects Psychol Med 1997 27 363 370 10.1017/S0033291796004382 9089829

[B62] Zigmond AS Snaith RP The hospital anxiety and depression scale Acta Psychiatr Scand 1983 67 361 370 10.1111/j.1600-0447.1983.tb09716.x 6880820

[B63] Cardol M Beelen A van den Bos GA de Jong BA de Groot I de Haan RJ Responsiveness of the Impact on Participation and Autonomy questionnaire Arch Phys Med Rehabil 2002 83 1524 1529 10.1053/apmr.2002.35099 12422319

